# Field Evaluation of Two Rapid Diagnostic Tests for *Neisseria meningitidis* Serogroup A during the 2006 Outbreak in Niger

**DOI:** 10.1371/journal.pone.0007326

**Published:** 2009-10-05

**Authors:** Angela M. C. Rose, Sibylle Gerstl, Ali E.-H. Mahamane, Fati Sidikou, Saacou Djibo, Laurence Bonte, Dominique A. Caugant, Philippe J. Guerin, Suzanne Chanteau

**Affiliations:** 1 Epicentre, Paris, France; 2 Centre de Recherche Médicale et Sanitaire (CERMES), Réseau International des Institut Pasteur, Niamey, Niger; 3 Médecins sans Frontières, Paris, France; 4 WHO Collaborating Centre for Reference and Research on Meningococci, Norwegian Institute of Public Health, Oslo, Norway; Norwegian Knowledge Centre for the Health Services, Norway

## Abstract

The Pastorex^®^ (BioRad) rapid agglutination test is one of the main rapid diagnostic tests (RDTs) for meningococcal disease currently in use in the “meningitis belt”. Earlier evaluations, performed after heating and centrifugation of cerebrospinal fluid (CSF) samples, under good laboratory conditions, showed high sensitivity and specificity. However, during an epidemic, the test may be used without prior sample preparation. Recently a new, easy-to-use dipstick RDT for meningococcal disease detection on CSF was developed by the Centre de Recherche Médicale et Sanitaire in Niger and the Pasteur Institute in France. We estimate diagnostic accuracy in the field during the 2006 outbreak of *Neisseria meningitidis* serogroup A in Maradi, Niger, for the dipstick RDT and Pastorex^®^ on unprepared CSF, (a) by comparing each test's sensitivity and specificity with previously reported values; and (b) by comparing results for each test on paired samples, using McNemar's test. We also (c) estimate diagnostic accuracy of the dipstick RDT on diluted whole blood. We tested unprepared CSF and diluted whole blood from 126 patients with suspected meningococcal disease presenting at four health posts. (a) Pastorex^®^ sensitivity (69%; 95%CI 57–79) was significantly lower than found previously for prepared CSF samples [87% (81–91); or 88% (85–91)], as was specificity [81% (95%CI 68–91) vs 93% (90–95); or 93% (87–96)]. Sensitivity of the dipstick RDT [89% (95%CI 80–95)] was similar to previously reported values for ideal laboratory conditions [89% (84–93) and 94% (90–96)]. Specificity, at 62% (95%CI 48–75), was significantly lower than found previously [94% (92–96) and 97% (94–99)]. (b) McNemar's test for the dipstick RDT vs Pastorex^®^ was statistically significant (p<0.001). (c) The dipstick RDT did not perform satisfactorily on diluted whole blood (sensitivity 73%; specificity 57%).

Sensitivity and specificity of Pastorex^®^ without prior CSF preparation were poorer than previously reported results from prepared samples; therefore we caution against using this test during an epidemic if sample preparation is not possible. For the dipstick RDT, sensitivity was similar to, while specificity was not as high as previously reported during a more stable context. Further studies are needed to evaluate its field performance, especially for different populations and other serogroups.

## Introduction

Every year during the meningitis season (approximately January–May), countries in the African meningitis belt are at risk from outbreaks of meningococcal meningitis. In order to effectively launch a mass vaccination campaign once an outbreak has been declared, the correct strain responsible for the outbreak must be identified. Traditional laboratory diagnostic methods for strain identification (such as culture and PCR) are expensive and can be complicated in terms of equipment and training needed. Often only a national or reference laboratory has the capacity to carry out these types of tests, as laboratory facilities in remote areas hit by an outbreak may be limited or non-existent.[1] Thus, samples are often transferred to the better-equipped central laboratories, which can be some distance away. The time taken for sample transfer, as well as the hot, dusty conditions experienced during the meningitis season, can lead to high levels of sample contamination, with inconclusive results.

### Pastorex^®^ rapid agglutination test

Since 2002, the World Health Organization has recommended pre-positioning rapid agglutination tests in peripheral laboratories prior to the meningitis season in countries in the African meningitis belt. One such test is the Pastorex^®^ rapid agglutination test (Bio-Rad Laboratores, Inc., Marne-la-Coquette, France), which can detect *Neisseria meningitidis* (*N. meningitidis*) serogroups A, C and Y/W135, as well as *N. meningitidis* serogroup B/*E.coli*, *Haemophilus influenzae*, *Streptococcus pneumoniae* and Group B *Streptococcus*. It is the main rapid agglutination test currently in use in the field for identification of *N. meningitidis* serogroup W135,[2] although it cannot distinguish *N. meningitidis* serogroup W135 from *serogroup* Y. The Pastorex^®^ test also carries certain constraints. Test reagents must be kept under cold chain (between +4°C and +8°C), and the manufacturer recommends heating and centrifuging cerebrospinal fluid (CSF) samples prior to using the test. The test kit contains reagents for 25 tests: once opened, all tests must be used within a month. Hence this test is not always practical for pre-positioning in peripheral health posts, but may be better used at district or regional hospital laboratory level. In epidemic situations at peripheral level, in the absence of equipment (centrifuge and/or heating device) or lack of trained laboratory personnel, there is sometimes little option other than to use the Pastorex^®^ test on unprepared CSF, i.e. without prior heating or centrifugation (Médecins sans Frontières (MSF) internal communication).

Several studies have investigated the performance of the Pastorex^®^ test under ideal laboratory conditions. Estimated sensitivity and specificity were 84% (95%CI 60–97) and 89% (76–96), respectively, for *N. meningitidis* serogroup W135,[2] while for serogroup A these were estimated at 88% (95%CI 85–91) and 93% (87–96), respectively.[3], [4] Some difficulties in reading the test results have been reported anecdotally by Pastorex^®^ test users (MSF internal communication), though mainly during outbreaks of serogroup W135. These could be due to user variation (e.g. differing interpretation of weak or borderline agglutination), or practical difficulties such as less than ideal kit conservation conditions in the field. In addition, the high ambient temperature and extremely dry conditions during meningitis season can lead to the drops of CSF and/or reagent drying out on the test strip before the results can be read. There may also be user error, such as the failure to follow the manufacturer-recommended sample preparation procedures of heating and centrifugation of the sample, mentioned above. To our knowledge, to date, investigation of the performance of this agglutination test under field conditions during an outbreak (i.e. using unprepared CSF) has not yet been conducted.[5]

The first aim of this study was therefore to observe whether the Pastorex^®^ test, when conducted under ‘field’ conditions (with no prior sample preparation) had similar sensitivity and specificity to this test when conducted following manufacturers' recommendations (i.e. with prior sample preparation; as reported previously[3], [4]).

### The dipstick rapid diagnostic test (RDT)

Recently, the Pasteur Institute in Paris and the Centre de Recherche Médicale et Sanitaire (CERMES) in Niamey, Niger developed a new dipstick RDT for the diagnosis of *N. meningitidis* serogroups A, W135, C and Y without prior sample preparation. This test exists as a duplex of two dipsticks (RDT1 and RDT2) in which RDT1 detects *N. meningitidis* serogroups A and W135/Y, while RDT2 detects *N. meningitidis* serogroups C and Y. An algorithm based on the results of the two dipsticks thus allows the detection of *N. meningitidis* serogroups A, C, W135 or Y. Under ideal laboratory conditions, the sensitivity and specificity for all serogroups were 100% when tested on reference strain cultures. Using frozen CSF samples, sensitivity was 94% for serogroup A, while specificity was 97%.[5] Results from 847 CSF samples received from suspected meningitis patients at CERMES in Niger from January 2005 to September 2006 showed that the dipstick RDT had a sensitivity of 89% (95%CI 84–93) and a specificity of 94% (95%CI 92–96).[1]

Our second aim was therefore to investigate whether this dipstick RDT, when conducted under ‘real’ field conditions, had a similar sensitivity and specificity to what has been previously reported for this new test under ‘ideal’ conditions. In addition, as the dipstick RDT, when commercialized, would have its main application at the peripheral health centre level, we wanted to directly compare results when the test was conducted on the same samples by (a) trained laboratory technicians in the laboratory vs (b) health post nurses in the field.

Finally, the fourth aim was to compare diagnostic accuracy of the dipstick RDT performed in the field on unprepared CSF with the Pastorex^®^ test performed under the same conditions.

As well as measuring performance of the dipstick RDT on CSF, we conducted a small sub-study to investigate how well this RDT performed on diluted whole blood samples.

Here we report on these evaluations of the Pastorex^®^ rapid agglutination test and the dipstick RDT, conducted in the field on unprepared CSF samples, as well as on diluted whole blood, during an epidemic in Niger in March-April 2006, using culture and/or PCR as the reference standard.

## Materials and Methods

### Study design, site and population

In previous evaluations, sensitivity and specificity of the Pastorex test for *N. meningitidis* serogroup A were found to be 88% and 93%, respectively, when the test was conducted following manufacturers' instructions for heat and centrifugation of sample prior to testing.[3], [4] We hypothesised that values for both sensitivity and specificity would be lower if the sample was not adequately prepared. We therefore calculated a sample size of 200, based on a prevalence of 70% among suspect cases presenting at health clinics, with targeted values for sensitivity and specificity of at least 70% (±8%) and 80% (±10%), respectively.

Patients were recruited prospectively between 27 February and 18 March 2006 in Niger during the outbreak of *N. meningitidis* serogroup A, which occurred in the Health District of Madarounfa in the Region of Maradi. The study setting was four peripheral health posts (Danissa, N'Yelwa, Safo and Serkin Yama), selected based on the following criteria: (a) having a high enough number of suspect cases during 1–2 weeks prior to the start of the study; (b) presence of health staff working at the health post who were trained in the lumbar puncture procedure and who could be trained to use the dipstick RDT; (c) availability of laboratory technicians to conduct the Pastorex^®^ tests on-site; (d) presence of a functioning refrigerator and freezer for stocking the Pastorex^®^ test reagents and freezing the CSF samples for transport, respectively. Both the National Ethical Review Committee of Niger and the Comité de Protection des Personnes (CPP “Ile de France XI”, France) approved the study prior to starting inclusions.

A suspect case was defined as a patient with clinical signs and symptoms of bacterial meningitis (see Appendix S1) presenting at one of the four health posts in the study. All suspect cases over the age of 2 months, and for whom an informed consent form had been signed, were eligible for inclusion. This form was read in the local language to the patient (or, for those who were under 15 years of age or in an altered state of consciousness, to the parent or other person accompanying the patient) by the attending health personnel at the health post. In each health post, a lumbar puncture was performed by a clinical officer in order to obtain a CSF sample, and blood samples were obtained by a finger-prick.

### Laboratory procedures

Approximately 4 ml of CSF was obtained from each eligible suspect case. A medical doctor from the research team supervised the lumbar puncture procedure in each health post at the start of the study and provided support and/or re-training as appropriate. Participating health posts were provided with Pastorex^®^ kits and dipstick RDT kits supplied by CERMES in Niamey (produced by the Institut Pasteur in Madagascar in December 2005). Bottles of trans-isolate (TI) media, for transport of the CSF for culture, were provided by the Norwegian Institute of Public Health (NIPH) in Oslo, and by CERMES.

The CSF was divided between 2 tubes, with one containing about 1 ml, which was immediately frozen for transport to CERMES for PCR. The health post nurse then inoculated the TI bottle with about 1 ml CSF from the second tube. Then 400 µl of CSF was removed from this tube and divided between two smaller tubes for immediate testing with the two dipstick RDTs by the health post nurse. The tube was later collected by the laboratory technician, for performing the Pastorex^®^ test on the remaining CSF. Where possible, the Pastorex^®^ tests were conducted in a separate room from the dipstick RDTs, often in batches.

If insufficient CSF was collected for all tests, the priority was to inoculate the CSF into TI for culture, or freeze the sample for PCR, or both.

Weekly during the study, frozen CSF samples and bottles of TI were transported to CERMES in Niamey, where both culture and PCR were performed, as well as a repeat of the dipstick RDTs. Briefly, after inoculating CSF at 36°C in chocolate blood medium and polyvitex, for 16–24 h, isolated colonies were re-inoculated on Muller Hinton media for a further 16–24 h. Colonies were then Gram stained and tested using API^®^ NH (Biomérieux). On the third day, isolates were serogrouped using rabbit anti-sera (BD Difco™) and tested for antibiotic resistance. PCR was performed using previously described methods.[4]–[8]

Different technicians performed culture and PCR, without knowledge of each other's results, and one technician (author SC) performed all the repeat dipstick RDTs in one batch at the end of the study. Results from all three tests were collated by CERMES and sent to Epicentre for analysis.

In addition to tests performed on CSF, 2 drops of blood (approximately 20 µl) were taken by the laboratory technician from a finger of each suspect case-patient and diluted with 180 µl phosphate-buffered saline in a small tube. A dipstick RDT was then conducted on these diluted whole blood samples in the field.

At the end of the study, a random 20% of CSF tubes was sent to the NIPH in Oslo for quality control by PorA gene-based nested PCR[9] followed by capsule gene-specific PCR on CSF positive with the PorA gene PCR.[10] In addition, 10 of the *N. meningitidis* serogroup A positive cultures were sent to the NIPH for PCR strain typing by MLST, PorA and FetA genotyping.

Treatment, free of charge, following the treatment protocol for epidemic meningococcal meningitis, was provided by MSF to all suspected patients at participating health posts. Treatment was based on clinical features, not on results of the rapid tests under evaluation.

### Analysis

#### Sensitivity and specificity

Data were entered onto EpiData^®^ Version 3.1 (EpiData, Odense, Denmark) and analysed initially using Stata™ Version 8.2 (StataCorp, College Station, TX, USA). The sensitivity and specificity were calculated, with 95% confidence intervals (95%CI). A reference standard of culture and/or PCR was selected as it had been used in previous evaluations of these tests,[2]–[5] and it was defined in the following way: positive samples were those having a positive result for either culture or PCR. Negative samples were those which were negative for both, or negative for one and not done or uninterpretable for the other. Samples which were contaminated in culture, and those with inhibited PCR results, were defined as uninterpretable for culture and PCR, respectively. Samples missing a result for any one of the tests under evaluation were excluded, so that we could directly compare sensitivity and specificity estimations across the different tests.

### Comparative analyses

#### Aims 1 and 2: Comparison of independent proportions

The sensitivity and specificity calculated for the Pastorex^®^ test were then compared with results published previously for this test on prepared CSF samples (Aim 1), and those for the dipstick RDT conducted in the field were compared with published results for this test conducted under ideal laboratory conditions (Aim 2). The t-test was used to compare proportions, with p-values<0.05 considered as statistically significant. In addition, the 95% CIs of the differences between these independent proportions were estimated, using Stata™ Version 10.0 (StataCorp, College Station, TX, USA), Newcombe's method.[11]

#### Aims 3 and 4: Comparison of paired samples

McNemar's test takes into account paired samples, which occurs when each sample has had more than one diagnostic test, as was the case in our study. This permitted us to compare results from the following pairs of samples: (a) dipstick RDT performed on CSF in the field vs the laboratory (Aim 3); (b) dipstick RDT vs Pastorex^®^ test, both performed on CSF in the field (Aim 4). Analysis (a) allowed us to examine whether the dipstick RDT performance varied by the conditions under which the test was performed, or by who performed it, while analysis (b) allowed us to compare the difference between two different tests when both were performed under similar conditions in the field.

## Results

**Figure 1:**
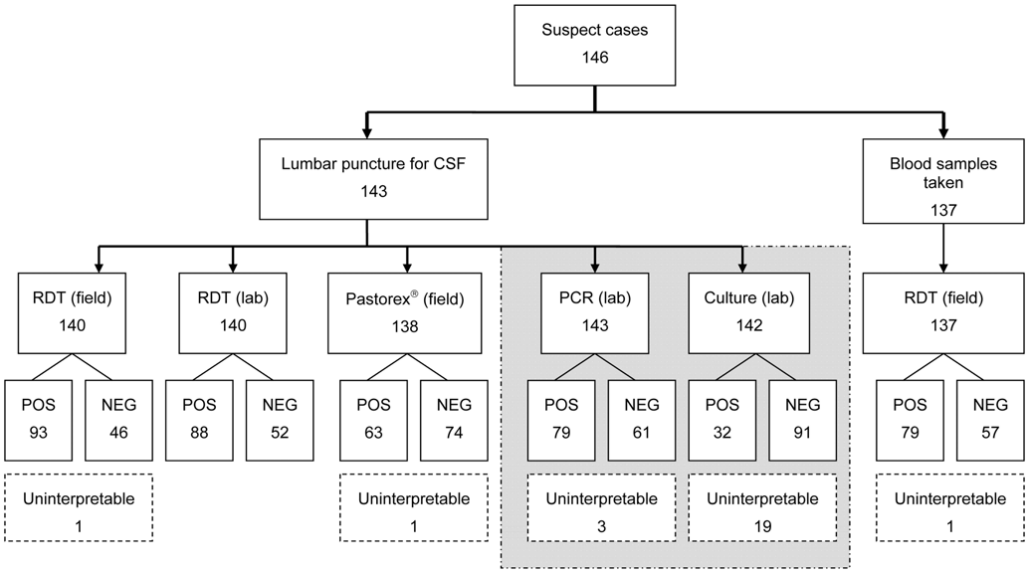
Schematic of all rapid and confirmatory diagnostic tests conducted on cerebrospinal fluid (CSF) and blood samples, with results obtained for each test. Shaded section shows confirmatory tests (‘reference standard’).

There were 146 suspect case-patients for whom lumbar punctures were conducted at the 4 health posts during this study. As three of these procedures failed to produce CSF, blood sampling was only attempted for the remaining 143 case-patients. Four died before blood could be collected and, for two others, the laboratory technician was not available for blood collection prior to starting treatment. Thus blood samples were collected from a total of 137 (94%) patients with suspected meningococcal disease (Figure 1).

**Figure 2:**
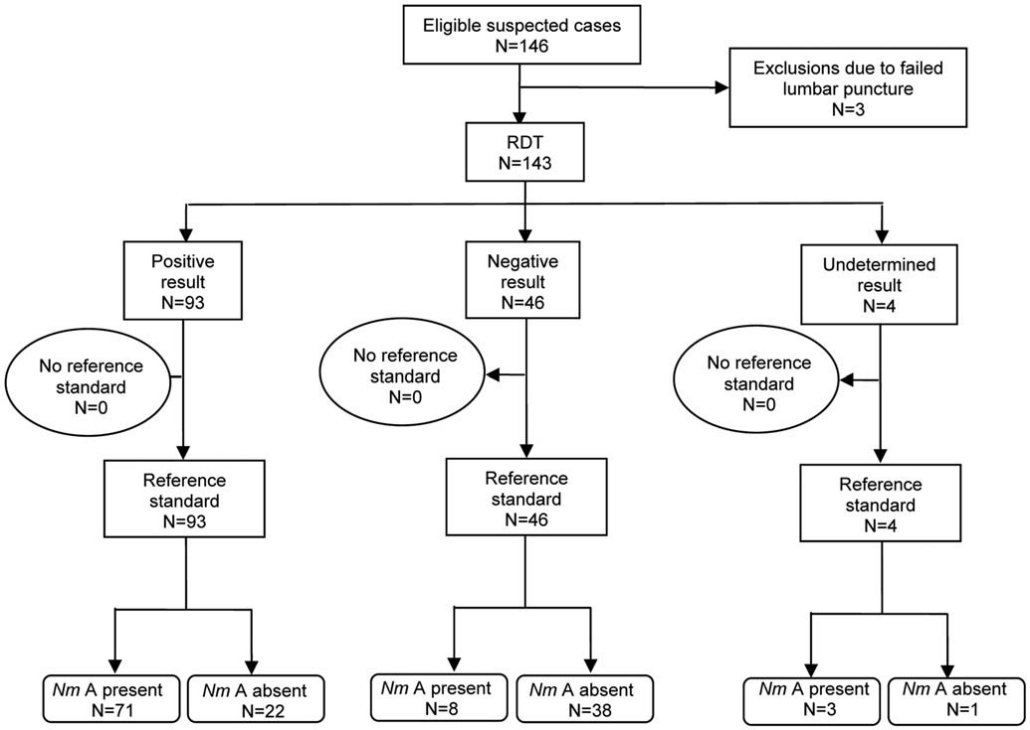
Flow diagram showing performance of the dipstick rapid diagnostic test (RDT) conducted in the field on unprepared cerebrospinal fluid, against a reference standard of culture and/or PCR, to diagnose *N. meningitidis* serogroup A (*Nm*A). (Note: “No reference standard” indicates those samples for which the reference standard result was undetermined or where there was not enough CSF remaining to conduct PCR).

**Figure 3:**
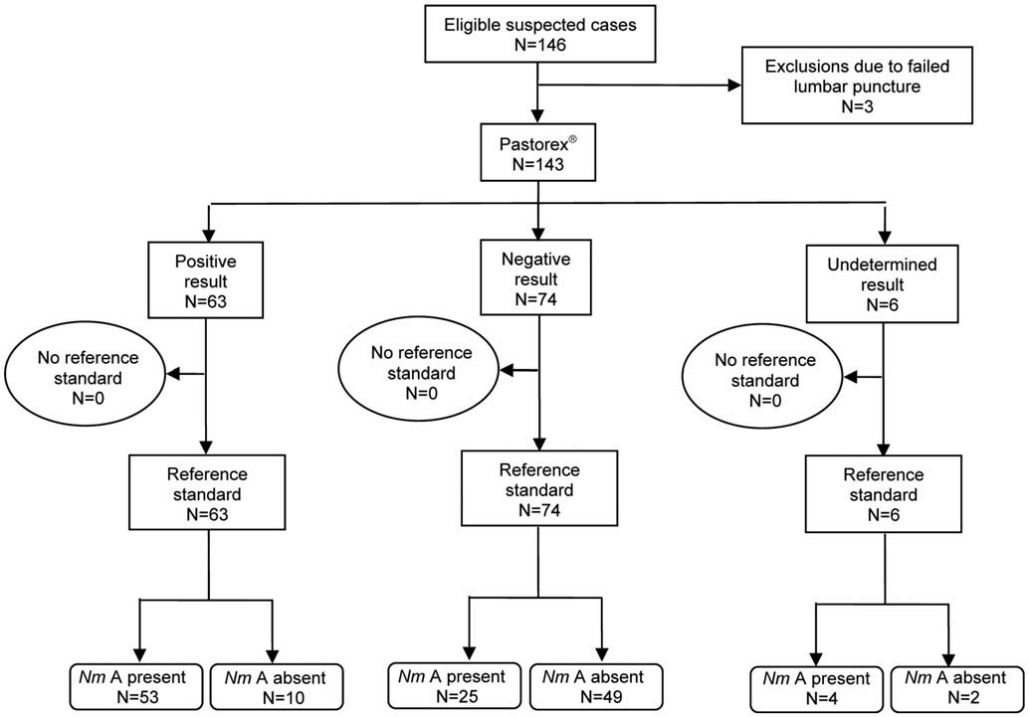
Flow diagram showing performance of the Pastorex^®^ test conducted in the field on unprepared cerebrospinal fluid, against a reference standard of culture and/or PCR, to diagnose *N. meningitidis* serogroup A (*Nm*A). (Note: “No reference standard” indicates those samples for which the reference standard result was undetermined or where there was not enough CSF remaining to conduct PCR).

Of the 143 CSF samples, 82 (57%) were positive by the reference standard of culture and/or PCR. Flow diagrams of the performance of the dipstick RDT and the Pastorex^®^ test, both conducted in the field on unprepared CSF, are shown in Figures 2 and 3, respectively. (Additional flow diagrams for performance of the dipstick RDT on unprepared CSF in the laboratory, and on undiluted whole blood in the field, are shown in Appendices S2 and S3, respectively.)

### Sensitivity and specificity

After exclusions due to insufficient CSF (n = 8) or no blood being collected (n = 6), or because of uninterpretable test results (n = 3), there were 126 samples with interpretable results for all CSF and blood tests (88%) (Table 1). Culture had the greatest proportion of uninterpretable results (19/142; 13%) compared with all other rapid and confirmatory tests conducted (≤2%). There were 73 samples confirmed positive by the reference standard out of the 126 samples having all test results; giving a prevalence during this period of the outbreak of 58% (95% CI 49–67).

**Table 1:** Comparison of results for each of the tests conducted vs the reference standard,* for the 126 samples having clear (either positive or negative) results for all tests.

		Reference standard*	Reference standard*	
Test site (fluid; test type†)		+	−	TOTAL
(1) Health post	**+**	65	20	85
(CSF; dipstick RDT)	**−**	8	33	41
(2) Laboratory	**+**	64	17	81
(CSF; dipstick RDT)	**−**	9	36	45
(3) Health post	**+**	53	23	76
(Blood; dipstick RDT)	**−**	20	30	50
(4) Health post	**+**	50	10	60
(CSF; Pastorex^®^)	**−**	23	43	66
	**TOTAL**	**73**	**53**	**126**

* Reference standard: culture and/or PCR.

† RDT: rapid diagnostic test.

An overview of the performance of each of the tests under evaluation in our study is provided in Table 2.

**Table 2:** Sensitivity and specificity, with 95% confidence intervals (95%CI) for cerebrospinal fluid (CSF) and blood samples from 126 suspect case-patients during the *Neisseria meningitidis* serogroup A outbreak in Niger; February–March 2006.*

Type (site) of RDT†	Sample type	Sensitivity % (95%CI)	Specificity % (95%CI)
RDT (field)	CSF	89 (80–95)	62 (48–75)
RDT (laboratory)	CSF	88 (78–94)	68 (54–81)
RDT (field)	Blood	73 (61–82)	57 (42–70)
Pastorex^®^ (field)	CSF	69 (57–79)	81 (68–91)

* Sensitivity and specificity were calculated versus a ‘reference standard’ of culture and/or PCR.

† RDT: dipstick rapid diagnostic test.

### Aims 1 and 2

The sensitivity and specificity of the Pastorex^®^ test on unprepared CSF samples were both lower than found previously using prepared CSF samples (Aim 1; Table 3). For sensitivity this difference was statistically significant using both comparative statistical tests: p<0.05 for the sensitivity in the current study compared with each of the prior studies;[3], [4] difference between the sensitivity in the current study and that found by Borel et al.[3] = 0.19 (95%CI 0.08–0.31); difference between the sensitivity in the current study and that found by Djibo et al.[4] = 0.18 (95%CI 0.07–0.30). Similarly, the specificity for the Pastorex® test was significantly lower (p<0.05) than in the prior studies, with a difference of 0.12 (95%CI 0.02–0.25) (Table 3).

**Table 3:** Results of statistical comparison tests conducted between the sensitivity and specificity of diagnostic tests performed in the field on unprepared CSF during an epidemic in Niger, February–March 2006, vs sensitivity and specificity results from the same diagnostic tests conducted in earlier studies, using prepared CSF.*

Diagnostic test	Study	Sensitivity %	(95%CI)	Statistical comparisons:† (1) p-value; (2) difference (95%CI)	Specificity %	(95%CI)	Statistical comparisons: (1) p-value; (2) difference (95%CI)
**Pastorex®**	**Current study**	**69**	**57–79**		**81**	**68–91**	
	Prior study: Ref 3	88	85–91	*(1) 0.00005*	93	90–95	(1) 0.02
				*(2) 0.19 (0.08–0.31)*			*(2) 0.12 (0.02–0.25)*
	Prior study: Ref 4	87	81–91	*(1) 0.00045*	93	87–96	(1) 0.01
				*(2) 0.18 (0.07–0.30)*			*(2) 0.12 (0.02–0.25)*
**RDT** ‡	**Current study**	**89**	**80–95**		**62**	**48–75**	
	Prior study: Ref 1	89	84–93	(1) 0.99	94	92–96	*(1) <0.00001*
				(2) 0.00 (−0.08–0.10)			*(2) 0.32 (0.20–0.46)*
	Prior study: Ref 5	94	96–96	(1) 0.13	97	94–99	*(1) <0.00001*
				(2) 0.05 (−0.01–0.15)			*(2) 0.35 (0.23–0.48)*

* Results from the current study are shown in bold type; statistically significant results are shown in italic type.

† (1) P-value for a comparison between the two proportions; (2) the difference between two independent proportions, with 95%CI, calculated using Newcombe's method (see text).

‡ RDT: dipstick rapid diagnostic test.

The sensitivity of the dipstick RDT under field conditions was similar to what has been previously reported when this test was performed on CSF under ideal laboratory conditions (Aim 2; Table 3). In our study, RDT sensitivity was 89% (95%CI 80–95) vs earlier reports of 89% (95%CI 84–93),[1] (p = 0.99); and 94% (95%CI 90–96),[5] (p = 0.13). The specificity in our study, however, was significantly lower than found previously (p<0.05).[1], [5] The differences between the specificity in the current study and those reported previously [1], [5] were 0.32 (95%CI 0.20–0.46) [1] and 0.35 (95%CI 0.23–0.48) [5].

### Aims 3 and 4

Table 4 shows a comparison of the results from the dipstick RDT performed on unprepared CSF in the field vs the same test performed in the laboratory. McNemar's test on these 137 paired sample results gave χ^2^ = 0.35 (p = 0.35), i.e. the dipstick RDT, when performed on the same samples under different conditions by different technicians does not give statistically significantly different results (Aim 3). However, the McNemar's test on paired results from the dipstick RDT vs the Pastorex^®^ test, both performed on unprepared CSF in the field (Table 5), gave a χ^2^ = 24.5 (p = 0.000001), which is statistically significant (Aim 4). The latter overall result indicates that the dipstick RDT is more likely than the Pastorex^®^ test to give a positive result, although it does not tell us which of these tests is more likely to give the *correct* result. For this, first we divided sample results into those which were positive, and those negative, by the reference standard. Then we performed McNemar's test again, for all positive and negative samples separately (Tables 6 and 7, respectively). Comparing the dipstick RDT to the Pastorex^®^ test for all positive samples gave χ^2^ = 14.2 (p = 0.0002), thus the dipstick RDT is more likely than the Pastorex^®^ test to correctly diagnose a positive sample as positive, and this is statistically significant. For all negative samples, χ^2^ = 10.3 (p = 0.0013); thus the dipstick RDT is more likely than the Pastorex^®^ test to incorrectly diagnose a negative sample as positive, and this difference is also statistically significant.

**Table 4:** Comparison of results obtained using the dipstick rapid diagnostic test (RDT) for diagnosis of *Neisseria meningitidis* serogroup A from unprepared CSF during an epidemic in Niger, February–March 2006: tests conducted in the laboratory vs on-site at the health post.

		RDT (laboratory)	RDT (laboratory)	
		+	−	Total
**RDT**	**+**	74	17	91
**(health post)**	**−**	12	34	46
	**Total**	86	51	137

**Table 5:** Comparison of results obtained from different tests used for diagnosis of *Neisseria meningitidis* serogroup A from unprepared CSF during an epidemic in Niger, February–March 2006: dipstick rapid diagnostic test (RDT) vs Pastorex^®^.

		RDT	RDT	
		+	−	Total
**Pastorex^®^**	**+**	60	2	62
	**−**	30	42	72
	**Total**	90	44	134

**Table 6:** Comparison of results from the Pastorex^®^ test vs the dipstick rapid diagnostic test (RDT) performed in the field on unprepared CSF during an epidemic in Niger, February–March 2006, for all positive samples by the reference standard of culture and/or PCR (N = 76).

		RDT	RDT	
		+	−	Total
**Pastorex^®^**	**+**	51	1	52
	**−**	17	7	24
	**Total**	68	8	76

**Table 7:** Comparison of results from the Pastorex^®^ test vs the dipstick rapid diagnostic test (RDT) performed in the field on unprepared CSF during an epidemic in Niger, February–March 2006, for all negative samples by the reference standard of culture and/or PCR (N = 58).

		RDT	RDT	
		+	−	Total
**Pastorex^®^**	**+**	9	1	10
	**−**	13	35	48
	**Total**	22	36	58

Thirty CSF samples were sent to the NIPH in Oslo for quality control. Of these, 19 (63%) were positive by PCR for *N. meningitidis* serogroup A, of which 18 (95%) had the same PorA sub-type (P1.20,9). When compared with PCR results from CERMES in Niger, the overall concordance between the two laboratories for these 30 samples was 83% (95%CI 65–94). The percentage agreement for PCR conducted at CERMES for those samples determined as ‘true positive’ by the NIPH was 87% (95%CI 75–98); while for those considered as ‘true negatives’, the agreement was 78% (95%CI 60–97). A representativity test for the 30 samples used in the quality control analysis revealed that these were representative of the samples collected in the field.

## Discussion

We show that the sensitivity and specificity of the Pastorex^®^ test and specificity of the dipstick RDT, performed on unprepared samples (i.e. without prior heating and centrifugation), in the field under epidemic conditions, are statistically significantly lower than previously reported values performed on prepared samples.[1], [3], [4], [5] We also found that the dipstick RDT performed at a central laboratory did not give significantly different results for sensitivity and specificity compared with testing under field conditions (Table 2). Finally, we show that the dipstick RDT, performed on the same samples, had higher sensitivity but lower specificity than the Pastorex^®^ test, and that these differences were statistically significant. Thus, under these conditions, the dipstick RDT is better at correctly ruling out disease (as it has a lower false negative rate), while the Pastorex^®^ test is better at confirming disease (having a lower false positive rate). The performance of the dipstick RDT on diluted whole blood samples was not satisfactory, as specificity was very low (57%). The sensitivity of this test on diluted whole blood, however, was similar to that of the Pastorex^®^ test conducted on unprepared CSF (73% vs 69%).

There were several limitations to our study. First of all, there were not enough ‘true negatives’ for us to be able to calculate acceptably accurate estimates of specificity for either of the rapid tests. Secondly, we only performed the Pastorex^®^ test on unprepared samples, so we can make no direct comparison with the test performed on prepared samples. Our results for the Pastorex^®^ test therefore have to be compared with those from previous studies, which is not ideal. In addition, our study had a smaller sample size than expected (146 vs 200), which occurred because, by the time we began the study, measures were already in place to bring the outbreak under control, and fewer patients with suspected meningococcal disease were presenting to health posts with symptoms. In particular, the sample size for patients with suspected meningococcal disease who were negative for *N. meningitidis* serogroup A was smaller than expected. These factors resulted in wide confidence limits (less precision) for both sensitivity and specificity. Finally, the PCR methods used in the laboratory in Niger (CERMES) and that in Oslo (NIPH) were based on different gene targets. In addition, the NIPH method involved a nested PCR, which appears to have somewhat greater sensitivity. These two points may explain the differences between the Niamey and Oslo PCR results in the quality control testing of a subset of samples.

Further estimates should be made using the dipstick RDTs in similar conditions, but with a larger sample size, in order to provide more precise estimates. It would also be useful to test the dipstick RDT performance on other populations and for other serogroups to assess the test's viability for widespread use throughout the meningitis belt. Clearly, to clarify the relative advantages and disadvantages of the Pastorex^®^ and dipstick RDT tests, further direct comparative studies must be conducted.

Even though the performance of the dipstick RDT on diluted whole blood was not as good as that on unprepared CSF, the test shows some promise, especially in terms of its sensitivity and ease of use. In particular, further development of this test is warranted if we are one day to avoid the difficult, often painful and potentially harmful lumbar puncture procedure. Even if the dipstick RDT, for either CSF or whole blood, never proves sufficiently accurate enough for bedside use (i.e. for physicians to make a decision on individual treatment), further development of this test should mean that the sub-Saharan African region could have a faster, cheaper and easier alternative for determination of a meningococcal meningitis outbreak. We strongly urge others to evaluate this test when investigating future outbreaks so that those involved in its development can have as much information as possible in order to further fine-tune this much-needed test.

Finally, because of logistical constraints during an epidemic, the Pastorex^®^ test is sometimes used without prior sample preparation. Based on our findings, i.e. low sensitivity and specificity, we recommend that this test should not be used during epidemics unless prior centrifugation and heating of CSF can be guaranteed. The use of hand-held centrifuges and battery-operated water-heaters should be explored in field settings to investigate whether, during epidemic conditions, they could serve as reliable alternatives to be used for CSF sample preparation prior to the Pastorex^®^ test.

## Supporting Information

Appendix S1 (0.03 MB DOC)

Appendix S2 Flow diagram showing performance of the dipstick rapid diagnostic test (RDT) against a reference standard of culture and/or PCR, to diagnose *N. meningitidis* serogroup A (*Nm*A) conducted in the laboratory on unprepared cerebrospinal fluid. (Note: “No reference standard” indicates those samples for which no reference standard result was obtained).
(0.33 MB TIF)

Appendix S3 Flow diagram showing performance of the dipstick rapid diagnostic test (RDT) against a reference standard of culture and/or PCR, to diagnose *N. meningitidis* serogroup A (*Nm*A) conducted in the field on undiluted whole blood. (Note: “No reference standard” indicates those samples for which no reference standard result was obtained).
(0.35 MB TIF)
