# Supplementary material for: Field Evaluation of Two Rapid Diagnostic Tests for Neisseria meningitidis Serogroup A during the 2006 Outbreak in Niger
Source: PLoS One. 2009 Oct 5;4(10):e7326. doi: 10.1371/journal.pone.0007326 (PMC2752163; doi:10.1371/journal.pone.0007326)
Supplement: Appendix S1 — (0.03 MB DOC) [file pone.0007326.s001.doc]

Appendix S1. Suspect case definition for bacterial meningitis.[12]

Adults and children over 12 months:

High fever of sudden onset (measured on arrival to the health post at ≥ 38.5°C) associated with at least one of the following signs:

- Stiff neck
- Petechial or puerperal rash

Children from 2 to 12 months:

High fever of sudden onset associated with at least one of the following signs:

- Bulging fontanelle
- Petechial or puerperal rash
